# Do physical activity, psychological health, and food group intake change in early pregnancy before and during COVID-19? A secondary analysis of cohort data from the Korea Nurses’ Health Study

**DOI:** 10.4069/whn.2025.03.05

**Published:** 2025-03-28

**Authors:** Chiyoung Cha, Jung Eun Lee, Jin-hui Han

**Affiliations:** 1 College of Nursing, Ewha Womans University, Seoul, Korea; 2 Ewha Research Institute of Nursing Science, Ewha Womans University, Seoul, Korea; 3 Department of Food and Nutrition, College of Human Ecology, Research Institute of Human Ecology, Seoul National University, Seoul, Korea

**Keywords:** Food group, Health behavior, Humans, Pandemics, Pregnancy

## Abstract

**Purpose:**

This study aimed to compare the physical activity, psychological health, and food group intake of women in their first trimester of pregnancy before and during the coronavirus disease 2019 (COVID-19) pandemic, and to assess these differences according to annual income levels.

**Methods:**

Using pregnancy survey data from the Korea Nurses’ Health Study, we analyzed 800 cases (381 pre-COVID-19, 419 COVID-19) for physical activity and psychological health, and 592 cases (296 pre-COVID-19, 296 COVID-19) for food group intake. Data were matched using 1:1 propensity score matching. Physical activity, psychological health, and food group intake were compared between periods and income groups.

**Results:**

During the pandemic, cycling (t=1.48, *p*=.003), aerobic exercise (t=0.98, *p*=.046), and light exercise (t=3.91, *p*<.001) increased while swimming decreased (t=–1.81, *p*<.001). Lower-income groups showed decreased running (t=–1.50, *p*=.004), swimming (t=–1.76, *p*<.001), and aerobic exercise (t=–1.10, *p*=.042), while higher-income groups showed increased participation in various physical activities. Depression scores decreased significantly in the lower-income group (t=–1.22, *p*=.022). Regarding food group intake, consumption of soups, stews (t=–1.63, *p*=.018), vegetables, kimchi, seaweed, and root (t=–1.60, *p*=.044) decreased during the pandemic. Lower-income groups showed decreased vegetable consumption (t=–1.43, *p*=.026) and alcohol intake (t=–1.34, *p*=.039), while higher-income groups showed increased alcohol consumption (t=1.29, *p*=.010).

**Conclusion:**

The COVID-19 pandemic differently affected health behaviors in early pregnancy based on income levels, highlighting socioeconomic disparities in maintaining healthy behaviors during public health crises. These findings suggest the need for tailored interventions considering income levels when promoting health behaviors among pregnant women during future public health crises.

## Introduction

The coronavirus disease 2019 (COVID-19) pandemic, caused by the severe acute respiratory syndrome coronavirus 2, has significantly impacted healthcare systems and personal health behaviors worldwide [1,2]. Among the vulnerable populations, pregnant women—especially those in early pregnancy—face unique challenges due to physiological and psychological adjustments [3,4]. The first trimester is a crucial period for fetal development and maternal adaptation, making any disruptions particularly concerning [4,5]. The additional stressors introduced by the pandemic may lead to changes in pregnant women’s health behaviors and mental well-being, potentially affecting both maternal and fetal health outcomes [4].

Physical activity during pregnancy is consistently associated with a wide array of health benefits, playing a crucial role in maintaining maternal and fetal well-being [6]. Regular exercise during this period has been shown to improve or maintain physical function, enhance cardiovascular health, assist in gestational weight management, and significantly reduce the risk of complications such as gestational diabetes and pre-eclampsia, while also contributing to improved psychological well-being [6]. However, the COVID-19 pandemic has caused unprecedented challenges in maintaining a regular physical activity routine, leading to a shift in exercise patterns among pregnant women. Studies have revealed that a significant proportion of expectant mothers experienced a substantial 64% decrease in physical activity levels coinciding with the onset of quarantine measures. Interestingly, however, a smaller segment of this population increased their activity levels by 15% [7]. These contrasts illustrate the complex interplay between pandemic-related restrictions and individual circumstances. Further, they suggest the need for tailored interventions and guidance that support pregnant women’s physical activity, even during a public health crisis.

Psychological health is an important factor during pregnancy, with 10% to 25% of pregnant women experiencing symptoms of anxiety and depression, which can increase the risk of preterm birth, postpartum depression, and behavioral disorders in children [8,9]. Prior research has shown a significant increase in anxiety and depression symptoms in pregnant women during the COVID-19 pandemic [8]. These increases can negatively impact not only the emotional state of pregnant women but also that of postpartum women, as well as fetal development [9]. Indeed, studies have reported a significant increase in maternal mental health issues, such as clinically relevant anxiety and depression, during this period [5].

Dietary factors play an important role in meeting the nutritional needs of both the mother and fetus, and adequate nutrition during pregnancy is essential for fetal growth and development as well as maternal health [10]. Disruptions in the food supply chain, economic pressures, and stress or confinement caused by the COVID-19 pandemic have led to changes in food availability, food choice, and eating habits, all of which can impact food intake [2,3]. Some women may have spent more time at home, allowing them more opportunities to prepare their meals, whereas others may have relied more heavily on processed or convenience foods [11]. Understanding these changes in nutrients, foods, and food groups is crucial to ensuring that pregnant women receive adequate nutrition during this critical time, despite global health concerns.

The COVID-19 pandemic has had a broad impact on the health of pregnant women, but the magnitude and nature of the impact vary considerably according to income level [12,13]. Pregnant women from higher-income groups may have been better able to exercise regularly and maintain a healthy diet owing to relatively better housing and the ability to work from home, whereas women from lower-income groups may have faced greater challenges in managing their health owing to economic instability and limited resources. Low-income pregnant women are more vulnerable to nutritional imbalances and subsequent fetal developmental problems due to limited access to food [12]. Previous studies on women’s income levels during pregnancy have also highlighted differences in stress levels [12,13], which also influence food group intake [12]. These disparities could have long-term implications for pregnancy outcomes and the health of the child, suggesting the need for differentiated approaches and support based on income level when designing pandemic response strategies.

Despite a growing body of research on the impacts of COVID-19, there is limited data on how the pandemic has specifically affected women in early pregnancy, particularly in terms of physical activity, psychological health, and dietary factors. This knowledge gap is significant because early pregnancy is a critical period for fetal development and maternal adaptation. Most studies have focused on the direct impact of COVID-19 on pregnancy outcomes or the general population’s response to the pandemic. However, the effects of the pandemic on critical health behaviors and psychological well-being during early pregnancy remain understudied.

Therefore, using pregnancy survey data from the Korea Nurses’ Health Study (KNHS), this study aimed to compare the physical activity, psychological health, and food group intake of female nurses in their first trimester of pregnancy before and during the COVID-19 pandemic, and to assess these the differences according to annual income.

## Methods

**Ethics statement:** This study was conducted using data from Korea Nurses’ Health Study, which were provided in an anonymized format without any sensitive information. As the study did not involve personally identifiable or sensitive data and was conducted anonymously, the requirement for obtaining informed consent was waived by the Institutional Review Board of Ewha Womans University (No. EIRB-ewha-202410-0023-01).

### Study design

This study is a secondary analysis of pregnancy survey data (2017–2023) from the KNHS, a cohort study of 20,613 female nurses of childbearing age (20–45 years) [14]. Participants who were pregnant at the time of the survey were asked to fill out the pregnancy survey, which aimed to describe the health and healthy lifestyle of pregnant women in their first trimester.

### Sample and sampling

This study included 1,322 women who completed the survey during their first trimester of pregnancy. We distinguished between the pre-COVID-19 and COVID-19 periods based on significant events and policy changes, using January 2020 as a reference point. We defined the COVID-19 period as beginning in January 2020, based on the World Health Organization’s declaration of a Public Health Emergency of International Concern on January 30, 2020 [15], and as the first confirmed case in South Korea occurred on January 20, 2020 [16].

**Figure 1.**
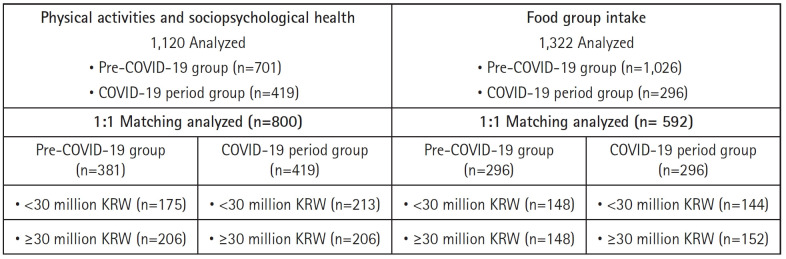
Flowchart of the study sample. COVID-19: Coronavirus disease 2019; KRW: Korean won.

Figure 1 describes the flowchart of the study population. We grouped the participants according to the pre-COVID-19 and COVID-19 periods from the survey items. For the survey items on physical activity and psychological health that asked about participants’ activities and feelings during the past month, 1,120 participants were identified between January 2017 and March 2022. We classified 701 participants in the pre-COVID-19 pandemic (January 2017 to December 2019) group and 419 in the COVID-19 pandemic (January 2020 to March 2022) group. Using the COVID-19 pandemic group with 419 participants, we matched data controlling for age, education, and annual income using 1:1 propensity score matching to control for differences in the characteristics of the two groups. We identified 381 pre-COVID-19 cases, resulting in a total of 800 cases.

For the survey items on foods and food groups that asked about participants’ food frequency during the past year, 1,322 participants were identified between January 2017 and March 2023. We classified 1,026 participants in the pre-COVID-19 (January 2017 to December 2020) group and 296 in the COVID-19 (January 2021 to March 2023) group. To minimize baseline differences and ensure comparability between the two groups, we conducted 1:1 propensity score matching based on age, education, and annual income. These variables were selected to create balanced groups and reduce potential confounding effects. Using the COVID-19 pandemic group with 296 participants, we matched an equal number of cases from the pre-COVID-19 group, resulting in a total of 592 cases for analysis.

### Variables

Sociodemographic factors (age, education, annual income), physical activity, psychological health, and food group intake were included as variables. Physical activity was measured using a translated tool adapted from the Nurses’ Health Study, which was conducted in the United States [17]. This scale consists of 11 physical activities: walking for exercise or work, jogging, running, cycling (including cycling machines), tennis (including squash and racquetball), swimming laps in a pool, other aerobic exercises (e.g., dancing, skiing, stepping), light exercise (e.g., yoga, stretching, calisthenics), other vigorous physical activities, and weight or resistance training. The average number of hours per week for each item is measured using a 10-point Likert scale: none, 1–4 minutes, 5–19 minutes, 20–59 minutes, 1 hour, 1–1.5 hours, 2–3 hours, 4–6 hours, 7–10 hours, and 11 or more hours. Higher scores (possible range, 10–110 hours) indicate a greater amount of physical activity.

Psychological health includes subjective health, fatigue, sleep quality, amount of sleep (in hours), anxiety, depression, and stress symptoms [14]. Subjective health was measured using one item on a 5-point Likert scale ranging from 1 (not at all) to 5 (very much). Higher scores (possible range, 1–5) indicated better subjective health.

Fatigue was measured using the 11-item Chalder Fatigue Scale [18] Korean version. This scale measures physical and mental fatigue and low energy levels on a 4-point Likert scale ranging from 0 (no problem) to 3 (very problematic). Higher scores (possible range, 0–33) indicate greater levels of fatigue. In this study, Cronbach’s alpha was .91.

Sleep quality was measured using the Jenkins Sleep Questionnaire [19] Korean version, which consists of four items evaluated on a 6-point Likert scale ranging from 1 (never) to 6 (every night). Possible scores range from 6 to 24. A score of 12 or more indicates frequent sleep disturbance and a score of 2 to 11 indicates occasional sleep disturbance. In this study, Cronbach’s alpha was .839. The amount of sleep was measured in hours using the item, “How many hours do you sleep on average?”.

Anxiety was measured using the 6-item Korean short-form of State-Trait Anxiety Inventory [20]. Measured on a 4-point Likert scale ranging from 1 (not at all) to 4 (very much), higher scores (possible range, 4–16) indicate higher anxiety. In this study, Cronbach’s alpha was .71.

Depression was measured using the Edinburgh Postnatal Depression Scale (EPDS) [21] Korean version, measured on a 4-point Likert scale ranging from 0 to 3. Higher scores (possible range, 0–30) indicate higher depression. Scores ≥10 are considered as clinically depressed in Korean women [22]. In this study, Cronbach’s alpha was .88.

Stress was measured using the 10-item Korean Perceived Stress Scale-10 (PSS-10) [23], rated on a 5-point Likert-type scale ranging from 0 (not at all) to 4 (very often). Higher scores (possible range, 0–40) indicate higher levels of stress. In this study, Cronbach’s alpha for the PSS-10 was 0.67, which falls within the acceptable range for exploratory research and short-form psychological scales [24]. While a reliability threshold of 0.70 or above is commonly considered acceptable, previous research suggests that values between 0.60 and 0.70 may be deemed appropriate in exploratory studies where the goal is hypothesis generation rather than confirmation. Given that PSS-10 is a brief measure with only 10 items, this reliability level is considered sufficient for capturing perceived stress levels while maintaining brevity and practicality [24].

Dietary information was assessed using the food frequency questionnaire developed by the Korea National Health and Nutrition Examination Survey to assess individuals’ nutrient intake [25]. The 112 food items were categorized into 10 groups: rice-noodles-dumplings, bread-rice cakes-cereals, soups-stews, beans-eggs-meat-fish, vegetables-kimchi-seaweed-root, dairy products, fruits, beverages, snacks, and alcoholic beverages. For each of the 112 food items, participants were asked to select one of nine categories based on the frequency of consumption during the past year: three times a day, twice a day, once a day, five to six times a week, two to four times a week, once a week, two to three times a month, once a month, and rarely. Each food category was analyzed as a continuous variable on a 9-point Likert scale ranging from 0 (rarely) to 8 (three times a day), and intake was calculated as the sum of the scores for each category.

### Data collection and analysis

The data were analyzed using IBM SPSS ver. 29.0 (IBM Corp., Armonk, NY, USA). Descriptive statistics were conducted to describe participants’ sociodemographic characteristics. Independent t-tests and chi-square tests were conducted to compare the differences between the groups according to the COVID-19 pandemic period and participants’ annual income status.

Income classification was initially grouped into 10 categories in the survey, ranging from an annual income of less than 20 million Korean won (KRW) to more than 60 million KRW. For analysis, these categories were reclassified into three broader income groups, which were less than 30 million KRW, 30 million to 45 million KRW, and more than 45 million KRW. At the time of data collection, the average annual income of Korean nurses was approximately 47.4 million KRW (32,521 US dollars [USD]) [26]. Given this economic context and the predefined income categories in the survey, 30 million KRW (approximately 20,835 USD) was used as a reference point to differentiate income groups in this study. This threshold was not based on a statistical mean or median but was chosen to reflect a meaningful division within the dataset, aligning with the existing income classification in the survey. Since the survey collected self-reported individual income rather than household income, the analysis was conducted using personal earnings. Additionally, because income was classified within predefined brackets, the study did not employ a continuous income variable for analysis.

## Results

### Differences in physical activity and psychological health before and during the COVID-19 pandemic

Table 1 describes the differences in physical activity and psychological health before and during the COVID-19 pandemic. For physical activity, scores for cycling (t=1.48, *p*=.003), other aerobic exercises (t=0.98, *p*=.046), and light exercise (t=3.91, *p*<.001) were statistically higher in the COVID-19 than the pre-COVID-19 group. By contrast, scores for swimming laps in the pool (t=–1.81, *p*<.001) were significantly lower in the COVID-19 group than in the pre-COVID-19 group. No statistical differences were found in the scores for psychological health between the two groups.

### Differences in physical activity and psychological health before and during the COVID-19 pandemic according to annual income level

Table 2 describes the differences in physical activity and psychological health based on participants’ annual income levels. For physical activity, participants with an annual income of less than 30 million KRW had statistically lower scores for running (t=–1.50, *p*=.004), swimming laps in a pool (t=–1.76, *p*<.001), and other aerobic exercises (t=–1.10, *p*=.042) during compared to before the pandemic. However, scores for light exercise were higher during than before the pandemic (t=1.01, *p*=.030) (Table 3). Participants with an annual income of 30 million KRW or more had higher scores for cycling (t=2.023, *p*<.001), other aerobic exercises (t=2.60, *p*<.001), light exercise (t=4.43, *p*<.001), other vigorous physical activities (t=1.74, *p*<.001), and weight training (t=2.03, *p*<.001) during compared to before the pandemic.

For psychological health, lower-income participants (<30 million KRW) had statistically lower scores for depression during the pandemic compared to before the pandemic (t=–1.22, *p*=.022). The mean EPDS score was 10.87 (standard deviation [SD], 6.06) before the COVID-19 pandemic and 10.16 (SD, 5.28) during the COVID-19 pandemic, indicating a slight decrease in depressive symptoms over time. Scores ≥10 are considered indicative of clinical depression in Korean women [14].

### Differences in food group intake before and during the COVID-19 pandemic

Table 3 describes the differences in participants’ food group intake before and during the COVID-19 pandemic. Scores for soups and stews (t=–1.63, *p*=.018) and vegetables, kimchi, seaweed, and root (t=–1.60, *p*=.044) were statistically lower in the COVID-19 group, indicating that the frequency of consuming these items was lower during the COVID-19 pandemic.

### Differences in food group intake before and during the COVID-19 pandemic according to annual income level

Table 4 describes the differences in food groups based on participants’ annual income levels. Lower-income participants scored lower for vegetables, kimchi, seaweed, and roots during the COVID-19 pandemic compared to before the pandemic (t=–1.43, *p*=.026), indicating that the frequency of consumption decreased during the pandemic. Alcoholic beverage consumption also differed by annual income level, i.e., lower-income participants scored lower alcoholic beverage intake during the pandemic (t=–1.36, *p*=.039) while the opposite was true for higher-income participants (t=1.29, *p*=.010)

## Discussion

Using data from the pregnancy survey of the KNHS, this study examined the impact of the COVID-19 pandemic on physical activity, psychological health, and food group intake among female nurses in their first trimester of pregnancy, with a particular focus on income-based differences. The findings reveal changes in health behaviors and highlight the critical role of socioeconomic factors in shaping these changes.

Regarding physical activity, the overall findings suggest that cycling, other aerobic exercises, and light exercise increased during the COVID-19 pandemic compared to before the pandemic, whereas swimming decreased. The increase in cycling is likely due to its nature as an outdoor, personal activity that allows for social distancing [27]. Similarly, an increase in other aerobic and light exercises, such as yoga and stretching, suggests a shift toward at-home or easily accessible forms of physical activity [11,27]. This shift is also consistent with global trends during the pandemic, where people adjusted their exercise routines in response to lockdowns and safety concerns [11]. The decrease in swimming is particularly noteworthy and can be directly linked to pool closures and concerns about virus transmission in public aquatic facilities. These findings emphasize how certain types of physical activity were disproportionately affected by pandemic-related restrictions. These shifts in physical activity patterns may reflect how COVID-19 has affected the capacity, opportunity, and motivation for different types of physical activity, resulting in reduced access to and participation in certain forms of physical activity, while activating other forms of physical activity more suited to pandemic restrictions [28].

The differences in physical activity by income level showed that lower-income group women showed a decrease in running, swimming, and other aerobic exercises, whereas those with higher income showed an increase in cycling, other aerobic exercises, light exercise, other vigorous physical activities, and weight training. These differences highlight the role of socioeconomic factors in health behaviors during the pandemic [12,13]. Although few studies have compared physical activity levels by income during the COVID-19 pandemic, higher-income individuals may have had more opportunities to engage in various physical activities due to better living conditions, access to home exercise equipment, and flexible work arrangements that allowed for more time to exercise. Conversely, lower-income individuals may have faced greater barriers to maintaining or adapting a physical activity routine due to limited space in their homes, less flexible work schedules, and financial constraints that limit the purchase of home exercise equipment. These disparities raise concerns that health inequalities may be magnified during public health crises. Future public health interventions should consider income-based differences in the ability to maintain healthy behaviors during difficult times. In particular, given that physical activity during early pregnancy is crucial for maternal and fetal health, the observed decrease in physical activity among lower-income women suggests a potential risk for adverse pregnancy outcomes, such as increased fatigue, poor mental health, and gestational complications. While some higher-income women reported engaging in activities such as cycling and weight training, it is important to recognize that certain exercises, particularly those that pose a risk of losing balance or excessive strain, may not be suitable during pregnancy. Strategies that promote physical activity during a pandemic or similar crises should include support that helps low-income populations maintain active lifestyles, with a special focus on pregnant women receiving appropriate guidance on safe exercise options to ensure their well-being.

Regarding psychological health, a statistically significant decrease in depression scores was observed in the low-income group, whereas no significant changes were found in the high-income group. This suggests that the decrease in depression among lower-income participants may have been influenced by factors such as reduced work-related stress or increased family support during lockdown [29]. In addition, no significant changes in anxiety levels, perceived stress, or sleep quality were observed in either income group. However, given the known challenges faced by healthcare workers during the COVID-19 pandemic, including increased workload and emotional strain, these findings should be interpreted with caution. While the results indicate relative stability in psychological health within this sample, it is possible that the professional background of the participants as nurses influenced their ability to cope with stress during the pandemic. These findings highlight the need for further research into the complex interactions between pregnancy, professional background, income level, and mental health in times of crisis.

Analysis of food group intake showing an overall decrease in the intake of soups and stews and vegetables, kimchi, seaweed, and root is likely due to changes in food purchasing behavior during communicable disease outbreaks such as the COVID-19 pandemic, reduced frequency of eating out, and changes in food preparation habits [11,30]. The decline in vegetables and seaweed consumption is of particular concern for pregnant women, as these are important sources of essential nutrients such as folic acid, iron, and iodine [31]. These trends may affect maternal and fetal health and highlight the need for pregnant women during public health crises, particularly outbreaks of infectious diseases.

Differences in food group intake were also observed by income level. Only low-income individuals showed a decrease in vegetables, kimchi, seaweed, and root intake and alcohol consumption during the COVID-19 pandemic. The decrease in vegetable consumption among low-income individuals is of particular concern and may reflect difficulties in obtaining or purchasing fresh produce during the pandemic [11,30]. Previous research indicates that overall spending on fresh and processed foods increased during the early phase of the pandemic due to a rise in home meal consumption [32]. However, as the pandemic progressed, spending on fresh foods, including vegetables, significantly declined [32]. Our study and previous studies suggest that while households initially prioritized grocery purchases, economic factors, changes in food prices, and shifting consumption habits may have contributed to a reduction in fresh produce intake over time, particularly among lower-income individuals.

The decrease in alcohol consumption in the low-income group and the increase in the high-income group is worth noting. Although the absolute numbers of alcohol consumption are relatively low, the upward trend in high-income pregnant women is particularly concerning given that the participants were not only in early pregnancy—a critical period for fetal development—but they were healthcare professionals who are expected to have a greater awareness of the risks of alcohol consumption during pregnancy. This may be related to the findings of decreased environmental rewards and increased depressive symptoms following social distancing during the pandemic, which may have led to increased drinking as a coping mechanism [33]. Given these findings, further research is needed to explore the factors influencing alcohol consumption among pregnant nurses, including workplace stress, shift work, and coping mechanisms. Understanding why some healthcare professionals engage in alcohol consumption despite their medical knowledge could inform targeted interventions. In addition, there is a need to continue and strengthen the education of healthcare professionals about the risks of drinking during pregnancy, particularly in the context of increased stress during infectious crises and the role of alcohol as a coping strategy. Healthcare providers should be aware that women, including those in the medical field, may turn to alcohol during stressful periods and should be prepared to provide appropriate interventions and support.

While this study provides valuable insights, it has several limitations. First, the sample was restricted to pregnant nurses, which was an intentional study design choice but may limit the generalizability of the findings to the broader population of pregnant women. Nurses, as healthcare professionals, may have different levels of health literacy, work-related stress, and access to health information compared to non-nurse pregnant women. Additionally, their work conditions, including shift work and exposure to healthcare environments, may uniquely influence their health behaviors and psychological well-being. Future research should explore whether similar patterns are observed in non-healthcare workers to understand the broader implications of these findings. Second, the study relied on self-reported data, which may introduce recall bias or social desirability bias, particularly in reporting sensitive information such as alcohol consumption or mental health symptoms. Using objective health measures and longitudinal tracking could help validate these findings in future studies.

In conclusion, this study demonstrates the complex interplay between the COVID-19 pandemic, socioeconomic status, and health behaviors among pregnant nurses. The findings highlight the importance of considering occupational factors and income disparities within healthcare professionals when evaluating maternal health outcomes. Given that nurses work in high-stress environments with variable shift schedules, workplace policies supporting mental well-being, flexible work hours, and structured maternal health programs could play a crucial role in promoting healthier pregnancies within this group.

Beyond nurses, these findings also have broader implications for non-healthcare pregnant women, particularly in terms of health education, public health interventions, and social support systems during public health crises. Policymakers should ensure that all pregnant women, regardless of occupation, have access to reliable health information, mental health support, and economic assistance programs to mitigate health disparities during future crises.

## Figures and Tables

**Table 1.** Differences in physical activity and psychological health before and during the COVID-19 pandemic (N=800)

Variable	Categories	Mean±SD or n (%)		*χ*²/*t* (*p*)
		Before COVID-19 (n=381)	During COVID-19 (n=419)	
Age (year)		32.52±2.94	32.63±3.04	–0.52 (.689)
	≤29	61 (16.0)	66 (15.8)	1.51 (.470)
	30–39	314 (82.4)	341 (81.4)	
	≥40	6 (1.6)	12 (2.8)	
Education	3-Year college graduate	187 (49.1)	209 (49.9)	0.13 (.938)
	4-Year college graduate	185 (48.5)	199 (47.5)	
	Master’s/doctorate	9 (2.4)	11 (2.6)	
Annual income (KRW)	<30 million	175 (45.9)	213 (50.8)	3.98 (.137)
	30 million–44.9 million	185 (48.6)	193 (46.1)	
	≥45 million	21 (5.5)	13 (3.1)	
Physical activity	Walking	6.32±2.41	6.08±2.30	–1.26 (.503)
	Jogging	1.48±1.17	1.53±1.20	0.53 (.370)
	Running	1.18±0.59	1.16±0.54	–0.66 (.194)
	Cycling	1.06±0.39	1.11±0.57	1.48 (.003)
	Tennis	1.05±0.29	1.04±0.42	–0.37 (.498)
	Swimming laps in a pool	1.10±0.57	1.04±0.34	–1.81 (<.001)
	Other aerobic exercises	1.09±0.43	1.12±0.57	0.98 (.046)
	Light exercise	2.04±1.43	2.50±1.84	3.91 (<.001)
	Other vigorous physical activities	1.10±0.47	1.12±0.55	0.53 (.248)
	Weight training	1.08±0.43	1.11±0.54	0.67 (.178)
Psychological health	Subjective health	2.45±0.79	2.38±0.77	–1.26 (.503)
	Fatigue	13.45±6.19	13.00±6.11	–1.02 (.952)
	Sleep quality	6.01±4.89	6.45±4.75	1.30 (.865)
	Amount of sleep (hour)	7.24±1.62	7.30±1.57	0.55 (.158)
	Anxiety	13.48±3.06	13.39±2.90	–0.41 (.122)
	Depression	10.65± 5.74	10.05± 5.32	–1.51 (.193)
	Stress	6.18±2.36	6.03±2.17	–0.92 (.104)

COVID-19: Coronavirus disease 2019; KRW: Korean won.

**Table 2.** Differences in physical activity and sociopsychological health between annual income groups before and during the COVID-19 pandemic (N=800)

Variable	Categories	<30 million KRW (n=388)			≥30 million KRW (n=412)		
		Mean±SD or n (%)		*χ*²/t (*p*)	Mean±SD or n (%)		*χ*²/t (*p*)
		Before COVID-19 (n=175)	During COVID-19 (n=213)		Before COVID-19 (n=206)	During COVID-19 (n=206)	
Age (year)		31.69±3.03	31.97±3.02	–0.91 (.164)	33.22±2.68	33.31±2.91	–0.31 (.372)
	≤29	47 (26.9)	47 (22.1)	1.9 (.374)	14 (6.8)	19 (9.2)	1.39 (.499)
	30–39	125 (71.4)	159 (74.6)		189 (91.7)	182 (88.4)	
	≥40	3 (1.7)	7 (3.3)		3 (1.5)	5 (2.4)	
Education	3-Year college graduate	103 (58.8)	120 (56.3)	1.41 (.493)	84 (40.8)	89 (43.2)	0.28 (.868)
	4-Year college graduate	71 (40.6)	89 (41.8)		114 (55.3)	110 (53.4)	
	Master’s/doctorate	1 (0.6)	4 (1.9)		8 (3.9)	7 (3.4)	
Physical activity	Walking	6.02±2.47	5.96±2.30	–0.25 (.278)	6.57±2.33	6.21±2.31	–1.62 (.886)
	Jogging	1.48±1.04	1.58±1.28	0.81 (.052)	1.49±1.27	1.48±1.12	–0.08 (.547)
	Running	1.22±0.64	1.14±0.50	–1.50 (.004)	1.15±0.55	1.18±0.57	0.53 (.346)
	Cycling	1.10±0.49	1.12±0.62	0.25 (.548)	1.03±0.26	1.11±0.53	2.02 (<.001)
	Tennis	1.08±0.39	1.05±0.52	–0.69 (.189)	1.03±0.17	1.04±0.78	0.43 (.373)
	Swimming laps in a pool	1.14±0.62	1.05±0.39	–1.76 (<.001)	1.07±0.53	1.03±0.27	–0.82 (.096)
	Other aerobic exercises	1.15±0.56	1.09±0.52	–1.10 (.042)	1.03±0.26	1.15±0.62	2.60 (<.001)
	Light exercise	2.18±1.45	2.34±1.74	1.01 (.030)	1.93±1.41	2.67±1.94	4.43 (<.001)
	Other vigorous physical activities	1.16±0.64	1.12±0.59	–0.61 (.274)	1.05±0.23	1.12±0.51	–1.74 (<.001)
	Weight training	1.15±0.60	1.11±0.56	–0.70 (.173)	1.02±0.18	1.10±0.52	–2.03 (<.001)
Psychological health	Subjective health perception	2.46±0.82	2.43±0.77	–0.39 (.301)	2.44±0.77	2.33±0.78	–1.47 (.901)
	Fatigue	12.85±6.44	12.85±6.29	–0.003 (.977)	13.95±5.93	13.16±5.94	–1.35 (.968)
	Sleep	5.81±5.04	6.55±4.76	1.49 (.441)	6.17±4.76	6.34±4.75	0.36 (.757)
	Amount of sleep	7.33±1.67	7.44±1.55	0.67 (.514)	7.16±1.57	7.15±1.57	–0.03 (.072)
	Anxiety	13.51±3.27	13.47±2.99	–0.14 (.118)	13.45±2.88	13.31±2.81	–0.49 (.468)
	Depression	10.87±6.06	10.16±5.28	–1.22 (.022)	10.46±5.46	9.94±5.39	–0.96 (.739)
	Stress	6.38±2.29	6.18±2.21	–0.87 (.653)	6.01±2.41	5.88±2.13	–0.59 (.136)

COVID-19: Coronavirus disease 2019; KRW: Korean won.

**Table 3.** Differences in food group intake before and during the COVID-19 pandemic (N=592)

Variable	Categories	Mean±SD or n (%)		*χ*²/*t* (*p*)
		Before COVID-19 (n=296)	During COVID-19 (n=296)	
Age (year)		33.04±3.03	33.27±3.19	–0.89 (.319)
	≤29	33 (11.2)	31 (10.5)	0.87 (.647)
	30–39	255 (86.1)	253 (85.5)	
	≥40	8 (2.7)	12 (4.0)	
Education	3-Year college graduate	149 (50.3)	146 (49.3)	0.23 (.890)
	4-Year college graduate	138 (46.6)	139 (47.0)	
	Master’s/doctorate	9 (3.1)	11 (3.7)	
Annual income (KRW)	<30 million	148 (50.0)	144 (48.7)	0.72 (.697)
	30 million–44.9 million	138 (46.6)	138 (46.6)	
	≥45 million	10 (3.4)	14 (4.7)	
Food group	Rice∙noodles∙dumplings	30.74±7.04	30.99±6.61	0.44 (.244)
	Bread∙rice cakes∙cereals	20.85±6.69	21.19±6.47	0.63 (.535)
	Soups∙stews	25.09±7.23	24.20±6.05	–1.63 (.018)
	Beans∙eggs∙meat∙fish	51.11±13.53	49.98±12.19	–1.07 (.155)
	Vegetables∙kimchi∙seaweed∙roots	65.80±21.44	63.09±19.68	–1.60 (.044)
	Dairy products	13.09±4.95	12.85±4.81	–0.61 (.281)
	Fruits	5.04±1.74	5.29±1.61	1.81 (.492)
	Beverages	13.55±5.31	13.28±5.04	–0.64 (.074)
	Snacks	15.89±6.27	15.72±6.41	–0.32 (.649)
	Alcoholic beverages	4.34±2.76	4.30±2.85	–0.18 (.978)

COVID-19: Coronavirus disease 2019; KRW: Korean won.

**Table 4.** Differences in food group intake between the annual income groups before and during the COVID-19 pandemic (N=592)

Variable	Categories	<30 million KRW (n=292)			≥30 million KRW (n=300)		
		Mean±SD or n (%)		*χ*²/t (*p*)	Mean±SD or n (%)		*χ*²/t (*p*)
		Before COVID-19 (n=148)	Before COVID-19 (n=144)		Before COVID-19 (n=148)	During COVID-19 (n=152)	
Age (year)		32.23±2.85	33.26±2.84	–0.10 (.965)	33.85±3.00	34.22±3.22	–1.02 (.409)
	≤29	24 (16.2)	22 (15.3)	0.05 (.976)	9 (6.1)	9 (5.9)	1.09 (.580)
	30–39	121 (81.8)	119 (82.6)		134 (90.5)	134 (88.2)	
	≥40	3 (2.0)	3 (2.1)		5 (3.4)	9 (5.9)	
Education	3-Year college graduate	86 (58.1)	79 (54.9)	3.24 (.198)	63 (42.6)	67 (44.1)	0.14 (.935)
	4-Year college graduate	62 (41.9)	62 (43.0)		76 (51.3)	77 (50.6)	
	Master’s/doctorate	0 (0)	3 (2.1)		9 (6.1)	8 (5.3)	
Food group	Rice∙noodles∙dumplings	30.41±7.42	30.89±7.17	0.57 (.391)	31.08±6.65	31.09±6.06	0.01 (.441)
	Bread∙rice cakes∙cereals	20.20±6.85	20.66±7.23	0.56 (.757)	21.50±6.49	21.70±5.64	0.28 (.204)
	Soups∙stews	24.15±7.41	23.84±6.57	–0.38 (.116)	26.03±6.93	24.53±5.52	–2.08 (.103)
	Beans∙eggs∙meat∙fish	50.98±13.91	49.78±12.62	–0.77 (.267)	51.24± 13.20	50.16±11.80	–0.74 (.391)
	Vegetables∙kimchi∙seaweed∙roots	64.64±23.24	61.00±19.96	–1.43 (.026)	66.95±19.49	65.07±19.23	–0.84 (.454)
	Dairy products	13.29±4.83	12.62±4.38	–1.25 (.210)	12.89±5.09	13.07±5.19	0.29 (.751)
	Fruits	4.91±1.82	5.15±1.63	1.16 (.195)	5.16±1.67	5.42±1.60	1.37 (.830)
	Beverages	13.86±5.54	13.76±4.99	–0.15 (.060)	13.24±5.06	12.82±5.05	–0.72 (.515)
	Snacks	16.20±6.75	15.86±6.47	–0.43 (.402)	15.58±5.76	15.59±6.37	0.01 (.836)
	Alcoholic beverages	4.55±3.24	4.08±2.74	–1.36 (.039)	4.12±2.16	4.51±2.94	1.29 (.010)

COVID-19: Coronavirus disease 2019; KRW: Korean won (1 million KRW was about 700 US dollars).
